# Associations Between High-Density Lipoprotein Subfraction Profiles and Heart Rate Response Following Submaximal Exercise

**DOI:** 10.3390/biology15131051

**Published:** 2026-07-01

**Authors:** Habib Al Ashkar, Nóra Kovács, Ilona Veres-Balajti, Ildikó Seres, György Paragh, Róza Ádány, Péter Pikó

**Affiliations:** 1 HUN-REN-UD Public Health Research Group, Department of Public Health and Epidemiology, Faculty of Medicine, University of Debrecen, 4032 Debrecen, Hungary; habib.al.ashkar@med.unideb.hu (H.A.A.);; 2 Doctoral School of Health Sciences, Faculty of Medicine, University of Debrecen, 4032 Debrecen, Hungary; 3 Department of Public Health and Epidemiology, Faculty of Medicine, University of Debrecen, 4032 Debrecen, Hungary; 4 Department of Physiotherapy, Faculty of Health Sciences, Institute of Health Sciences, University of Debrecen, 4028 Debrecen, Hungary; 5 Institute of Internal Medicine, Faculty of Medicine, University of Debrecen, 4032 Debrecen, Hungary; 6 National Laboratory for Health Security, Center for Epidemiology and Surveillance, Semmelweis University, 1089 Budapest, Hungary; 7 Institute of Preventive Medicine and Public Health, Semmelweis University, 1089 Budapest, Hungary

**Keywords:** HDL subfractions, lipid metabolism, lipoprotein heterogeneity, post-exercise heart rate response, heart rate recovery

## Abstract

The association of HDL and its subfractional profile with cardiovascular health, particularly atherosclerosis, is well established; however, its association with post-exercise heart rate response remains underexplored. This cross-sectional study investigated whether HDL subfraction distribution is associated with post-exercise heart rate dynamics. We analyzed 304 adults, stratifying HDL into ten subfractions and 3 subclasses using the Lipoprint^®^ system. Heart rate was measured at rest (HR_rest_), immediately after the YMCA 3-min step test (HR_aft_), and during recovery (HR_5min_ and HR_10min_) to calculate ΔHR. Multiple regressions were applied with False Discovery Rate correction. Participants with a more favorable post-exercise heart rate profile exhibited higher ApoA-I levels and favorable lipid ratios. Subfractions spanning the large and intermediate ranges (HDL-3 to HDL-5) were inversely associated with HR_aft_, HR_5min_, and ΔHR. In contrast, smaller, lipid-poor subfractions (HDL-7 to HDL-10) were associated with higher heart rates and a less favorable post-exercise response. Total HDL-C and subclass-level concentrations showed no significant association. These findings suggest that HDL particle size distribution may provide exploratory insight into exercise-related cardiovascular responses beyond conventional lipid metrics. Although limited by the use of a submaximal field test and manual heart rate assessment, these results support further investigation of HDL subfraction profiling in relation to post-exercise heart rate dynamics.

## Simple Summary

How rapidly the heart rate returns to normal after physical exertion is a practical indicator of overall cardiovascular health. A faster heart rate response is generally associated with better cardiovascular adaptability, while delayed responses may reflect less favorable cardiovascular function. This study investigated whether the specific size distribution of high-density lipoprotein (HDL) is linked to this dynamic heart rate response. While overall HDL levels are routinely measured in clinical settings, HDL actually comprises numerous different particle sizes with varying biological functions. By analyzing the blood profiles and exercise responses of 304 adults, this research found that a more favorable HDL subfraction composition, characterized by a higher relative proportion of large and intermediate HDL particles and a lower relative proportion of small HDL particles, was associated with more favorable post-exercise heart rate responses. These findings suggest that HDL particle distribution, rather than total HDL-C, may be related to exercise-related heart rate dynamics beyond standard cholesterol tests.

## 1. Introduction

The cardiovascular system’s ability to maintain homeostasis requires dynamic physiological responses to external stressors, such as physical exercise [1,2]. Heart rate dynamics following acute exercise are widely used as a non-invasive indicator of cardiovascular response to physical stress [3]. Elevated resting HR (HR_rest_) and reduced HR variability (HRV) are associated with vascular stress, reduced exercise capacity, and poorer overall cardiovascular outcomes [4,5,6].

Moreover, heart rate responses during and immediately after submaximal exercise are practical measures of post-exercise cardiovascular recovery [7,8]. More rapid heart rate recovery after exertion has been associated with more favorable exercise-related cardiovascular responses and may have prognostic value for future cardiovascular and metabolic health [7,9,10,11].

Lipid dysregulation is a major contributor to chronic cardiometabolic disease, particularly atherosclerotic cardiovascular disease (ASCVD) [12]. Standard lipid panels, including total cholesterol (TC), triglycerides (TGs), low-density lipoprotein cholesterol (LDL-C), and high-density lipoprotein cholesterol (HDL-C), remain essential components of cardiovascular risk assessment. HDL is generally associated with a lower ASCVD risk, and this relationship is partly linked to reverse cholesterol transport and cholesterol efflux capacity, which help limit macrophage foam cell accumulation [13,14].

HDL is now recognized as a structurally and functionally heterogeneous population of particles with diverse biological activities, including antioxidant, anti-inflammatory, and vasodilatory effects [15,16,17]. Larger HDL particles, enriched in apolipoprotein A-I (ApoA-I) and sphingosine-1-phosphate (S1P), have been reported to support endothelial NO synthase (eNOS) signaling and vascular function [18,19,20]. This relationship may be relevant to cardiovascular recovery following physical exertion [10,11]. In contrast, smaller, lipid-poor HDL subfractions are more susceptible to oxidative modification and are frequently associated with systemic inflammation and less favorable cardiometabolic profiles [21,22]. Recent proteomic studies further suggest that specific HDL subfractions are related to subclinical atherosclerosis and impaired metabolic health.

Although an unfavorable heart rate response to physical activity and dyslipidemia are recognized cardiovascular risk factors, the specific relationship between HDL particle size distribution and post-exercise heart rate recovery remains underexplored. This cross-sectional study aimed to investigate exploratory associations between detailed HDL subfraction profiles quantified via the Lipoprint^®^ system, traditional lipid parameters (TC, TG, LDL-C, ApoA, ApoB, and derived ratios), and multiphase heart rate indices following the YMCA 3-min step test. We hypothesized that a predominance of HDL subfractions in the large and intermediate ranges would be associated with a more favorable post-exercise heart rate response, providing exploratory insight into the relationship between HDL subfraction profiles and exercise-related heart rate dynamics.

## 2. Materials and Methods

### 2.1. Study Design and Populations

A complex health survey was conducted in 2018 in two northeastern Hungarian counties (Hajdú-Bihar and Szabolcs-Szatmár-Bereg). A full description of the design and data collection can be found in a previous paper [23]. In brief, the cross-sectional survey consisted of three pillars (i.e., a questionnaire survey, physical examinations, and laboratory tests). A total of 832 participants aged 20–64 years were randomly recruited, 417 from the Hungarian general (HG) population (232 women and 185 men) and 415 from the Roma population (307 women and 108 men). The ethnicity of the participants was self-reported. The questionnaire was based on the second wave of the European Health Interview Survey (EHIS) [24]. The questionnaire collected demographic characteristics (e.g., age, sex, and ethnicity); socioeconomic information; and health-related data (e.g., medication use). It has been extended to include the long version of the International Physical Activity Questionnaire (IPAQ), which measures physical activity across domains and intensities. Fasting blood samples were collected to conduct routine laboratory tests, including TC, TG, LDL, HDL-C, and fasting glucose levels. As part of the physical examination component of the survey, anthropometric data, including weight and height, were collected to calculate body mass index (BMI), and waist circumference was also measured. Blood pressure (BP) measurements were taken, and the YMCA 3 min step test was performed for each participant.

Samples were selected from the original HG and Roma populations for HDL subfraction analysis according to the following criteria. Participants with missing data (20 individuals from the HG sample and 47 from the Roma sample) and those receiving lipid-lowering treatment (27 from the HG sample and 43 from the Roma sample) were excluded. The remaining 695 subjects (370 from the HG and 325 from the Roma) were divided into two subgroups based on their lipid profiles. The healthy lipid profile group (126 HG and 87 Roma) included individuals with normal HDL-C (≥1.03 mmol/L in men and ≥1.29 mmol/L in women), TG (<1.7 mmol/L), TC (<5.2 mmol/L), and LDL-C (<3.4 mmol/L) levels, whereas the abnormal lipid profile group (244 HG and 238 Roma) included individuals with at least one abnormal lipid parameter.

A total of 277 subjects with abnormal lipid profiles (115 from HG and 162 from Roma) and 100 subjects with healthy lipid profiles (25 men and 25 women from each population) were selected for HDL subfraction analyses. The participants of the present study were selected from this HDL subfraction sample population.

**Figure 1:**
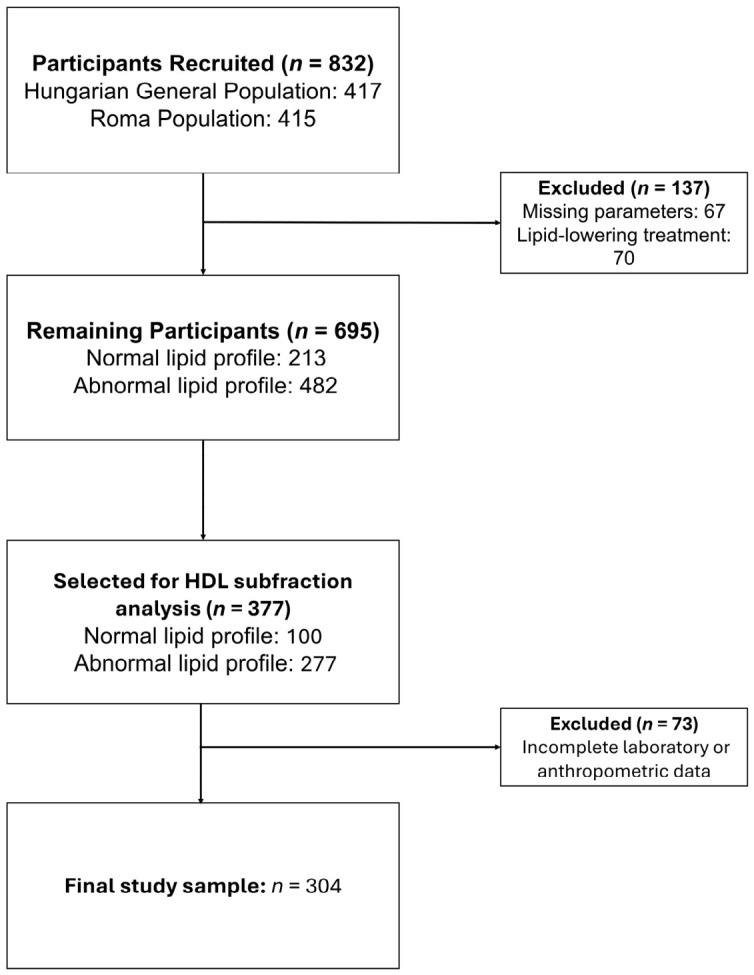
The flowchart illustrates the sample selection process for the study.

In our current study, participants with incomplete laboratory and/or anthropometric data were excluded (*n* = 73), and a total of 304 participants were selected. Figure 1 summarizes the sample selection process.

### 2.2. Measurement of Physical Activity and Heart Rate in Response to Physical Activity

The long form of the IPAQ was used to measure participants’ physical activity levels [25] by assessing time spent in light, moderate, and vigorous physical activities over the previous 7 days across various domains, including work, transportation, leisure time, domestic and gardening activities, and time spent sitting. Using the standardized IPAQ scoring protocol, only activities lasting more than 10 min were recorded. The results were then used to calculate the MET-min/week for each participant.

The YMCA 3 min step test, a well-established submaximal field test, was used to assess exercise-related heart rate responses. This protocol provides a practical, standardized physiological load suitable for large population-based surveys [26]. Each test began with the participant seated in a chair in a quiet room for a 2 min rest period. Participants were instructed to step up and down on a 30 cm step or bench 72 times within 3 min, maintaining a pace set by a metronome at 96 beats per minute (4 beats per step cycle, corresponding to 24 steps per minute, or 72 steps/3 min). After completing the test, the participants immediately sat down and remained still for 5 s.

Heart rate was measured at four time points during the YMCA 3 min step test: one minute before the test while at rest (HR_rest_), immediately after the test (HR_aft_), 5 min (HR_5min_), and 10 min (HR_10min_) post-test. Heart rate was manually measured using radial artery palpation for 60 s by the participating general practitioner or their assistant according to the standard clinical procedure in each practice. Although manual palpation does not provide continuous beat-to-beat data, counting the radial pulse for a full 60 s is a practical and widely accepted method of obtaining heart rate measurements in population-based field studies [27,28]. The difference between HR_rest_ and HR_aft_ was calculated and defined as delta heart rate (ΔHR), with lower values indicating a more favorable post-exercise heart rate response [9].

According to the YMCA step test classification criteria [29], the study population was stratified into three groups: very poor/poor (*n* = 79), average/above/below average (*n* = 133), and good/excellent (*n* = 92). This classification is based on standardized normative values, with post-exercise heart rate evaluated against 10-year age intervals with separate criteria for men and women. The detailed age- and sex-adjusted heart rate cut-offs used for this categorization are provided in Supplementary Table S1.

Participants in the very poor/poor category were considered to have an unfavorable heart rate response to exercise, whereas those in the good/excellent category were classified as having a favorable response. For logistic regression analysis, ΔHR was additionally dichotomized into a binary outcome variable (ΔHR_Bi_), distinguishing between unfavorable and favorable heart rate response profiles.

To further assess the heart rate response to physical activity, the age-related maximum heart rate (*HR_max_*) was calculated using the following formula [30]:

(1) $${HR}_{max}=220-age$$

To assess and compare target heart rate zones, *HR_max_* was expressed as a percentage using the following formula [31]:

(2) $${HR}_{max\%}=\frac{{HR}_{aft}}{\left(220-age\right)}\times 100$$

The reference group consisted of participants with an *HR_max_*% of 64% or below, whereas the adverse group included those with an *HR_max_*% exceeding 76% [32].

### 2.3. Analysis of Lipids and HDL Subfractions

Fasting blood samples were collected, and all lipid and apolipoprotein measurements, including HDL subfraction profiling, were performed from fresh serum samples. Specifically, total cholesterol and triglyceride concentrations were measured using the enzymatic colorimetric method (GPO-PAP, Modular P-800 Analyzer; Roche/Hitachi, Basel, Switzerland). HDL-C and LDL-C levels were assessed by homogeneous enzymatic colorimetric assays (Roche HDL-C plus 3rd generation and Roche LDL-C plus 2nd generation, Basel, Switzerland). Apolipoprotein concentrations were determined by immunoturbidimetric assays: ApoA-I using Tina-Quant ApoA-I Version 2 and ApoB using Tina-Quant ApoB Version 2 (Roche, Basel, Switzerland). The ApoB assay specifically quantifies the full-length ApoB-100 isoform present in LDL, VLDL, IDL, and Lp(a) particles; throughout the manuscript, ApoB refers to ApoB-100.

HDL subfraction profiling was performed using the Lipoprint^®^ HDL Subfractions Testing System (Quantimetrix Corporation, Redondo Beach, CA, USA), which separates HDL into 10 electrophoretic bands grouped into large HDL (HDL-L; subfractions 1–3), intermediate HDL (HDL-I; subfractions 4–7), and small HDL (HDL-S; subfractions 8–10). The cholesterol content of each HDL subfraction was expressed in mmol/L, along with the percentage of each HDL subfraction’s cholesterol concentration relative to total HDL-C. This technique employs high-resolution 3% polyacrylamide gel tubes for electrophoretic separation, following the manufacturer’s instructions, as described in more detail previously [33].

### 2.4. Statistical Analysis

A priori power analysis was conducted using G*Power 3.1.9.7 to determine the minimum sample size required for the primary analysis of heart rate outcomes. Assuming a medium effect size (f^2^ = 0.15), α = 0.05, and 12 predictors in the model, the analysis indicated a required sample size of 127 participants for 80% power and 184 participants for 95% power. Our final sample exceeded the thresholds, indicating that the study was adequately powered to detect statistically meaningful associations between HDL subfractions and heart rate outcomes.

The Shapiro–Wilk test for normality was used to determine the distribution of the quantitative data. Templeton’s two-step method was used to normalize the data where needed [34]. The Mann–Whitney U and Pearson’s chi-square tests were used in comparison analyses. The Jonckheere–Terpstra trend test [35] was used to assess the trend across the groups.

Multivariable logistic and linear regression analyses were performed to examine the association between the HDL subfraction profile and HR. All regression analyses were adjusted for age, BMI, ethnicity, sex, smoking status, diastolic and systolic blood pressure, glucose level, leisure-time physical activity in MET-min/week, and treatment for hypertension and diabetes. Variance inflation factors (VIFs) were calculated to assess multicollinearity; all predictors had VIF values below 5, indicating no substantial multicollinearity. All analyses were performed using SPSS software v30.0 (IBM, Armonk, NY, USA). Figures were created using GraphPad Prism version 8.0.0 for Windows (GraphPad Software, San Diego, CA, USA).

To control for type I errors, the Benjamini–Hochberg approach [36] was used to adjust *p*-values for multiple tests of the same dependent variable; adjusted *p*-values < 0.05 were considered significant.

## 3. Results

### 3.1. Baseline Characteristics of the Study Population

The study population was stratified into three groups based on heart rate response to exercise (ΔHR): very poor/poor, above average/average/below average, and good/excellent. Age increased progressively across the groups, with mean values of 37.08 years, 40.53 years, and 41.99 years, respectively (*p* for trend = 0.014). In contrast, ΔHR decreased markedly across the groups (66.68, 26.96, and 15.88; *p* for trend < 0.001), as did maximum HR expressed as a percentage of age-predicted *HR_max_* (80.52%, 59.22%, and 49.52%; *p* for trend < 0.001).

The prevalence of overweight and obesity also declined significantly across the groups (74.68%, 63.16%, and 56.52%; *p* for trend = 0.021), as did the proportion of individuals with elevated diastolic blood pressure (18.99%, 13.53%, and 5.43%; *p* for trend = 0.007). Leisure-time physical inactivity decreased across the groups as well (39.24%, 29.32%, and 20.65%; *p* for trend = 0.026). By contrast, no significant differences were observed for ethnicity, sex distribution, smoking status, elevated systolic blood pressure, antihypertensive treatment, antidiabetic treatment, fasting glucose level, or reduced HDL-C levels. For more details, see Table 1.

### 3.2. Comparison of the Lipid Profile Across ΔHR Groups

Trend analysis across ΔHR categories showed that HDL-C increased progressively with improving ΔHR (*p* = 0.014), accompanied by a significant increase in ApoA-I concentrations (*p* = 0.018). In contrast, the total cholesterol/HDL-C ratio (*p* = 0.010), LDL-C/HDL-C ratio (*p* = 0.008), and ApoB/ApoA-I ratio (*p* = 0.009) decreased significantly across the groups. TG/HDL-C also showed a downward trend, although this did not reach statistical significance (*p* = 0.063). TG, total cholesterol, LDL-C, ApoB, and LDL-C/ApoB did not differ significantly across the ΔHR groups (all *p* > 0.05). For more details, see Table 2. Baseline lipid and apolipoprotein profile distributions across ΔHR categories, stratified by sex, are detailed in Supplementary Table S2.

In the adjusted regression models, the ApoB/ApoA-I ratio was significantly associated with HR_5min_ (β = 7.692; *p* = 0.049) and ΔHR_Bi_ (β = 4.731; *p* = 0.033). The LDL-C/HDL-C ratio was also significantly associated with ΔHR_Bi_ (β = 1.435; *p* = 0.033). No other lipid parameters showed significant associations with the HR indices examined in the adjusted models (see Supplementary Table S3).

### 3.3. Comparison of HDL Subfractions and Subclasses Across ΔHR Groups

**Figure 2:**
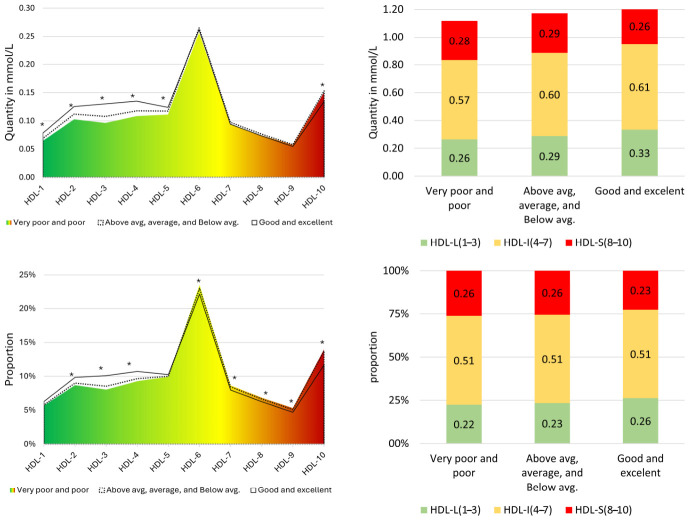
Composition and trend analysis of the HDL subfraction and subclass profiles in mmol/L and % by the ΔHR groups (very poor and poor; above average, average, and below average; and good and excellent); *: *p* < 0.05.

A significant positive trend (*p* < 0.05) was observed with the proportion of HDL-2 to HDL-4 subfractions across the 3 groups, while a significant negative trend was observed for HDL-6 to HDL-10 subfractions. Similarly, the concentrations of HDL-1 to HDL-5 subfractions (in mmol/L) increased significantly with improving ΔHR, while the HDL-10 concentration decreased significantly across the groups. Figure 2 visualizes these trends, highlighting both subfraction-level changes and corresponding shifts in HDL subclasses across ΔHR categories.

### 3.4. Association of HDL Subfraction Profile with ΔHR

**Figure 3:**
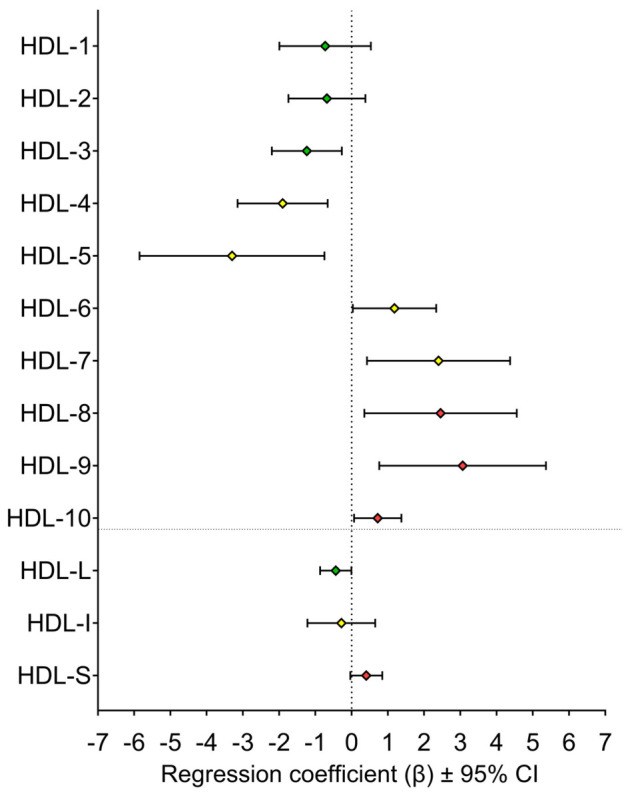
Association of HDL subfraction and subclass percentages with ΔHR. Colors represent the different HDL particle subclasses: green for large HDL (HDL-1 to HDL-3 and HDL-L), yellow for intermediate HDL (HDL-4 to HDL-7 and HDL-I), and red for small HDL (HDL-8 to HDL-10 and HDL-S). The vertical dotted line marks a regression coefficient of zero. The horizontal dotted line separates the 10 individual subfractions from the 3 aggregated subclasses.

The proportions of HDL-3 (β = −1.238; *p* = 0.030), HDL-4 (β = −1.904; *p* = 0.030), and HDL-5 (β = −3.301; *p* = 0.037) were significantly inversely associated with ΔHR. In contrast, HDL-7 (β = 2.397; *p* = 0.036), HDL-8 (β = 2.454; *p* = 0.037), HDL-9 (β = 3.063; *p* = 0.045), and HDL-10 (β = 0.720; *p* = 0.044) showed a significant positive association. However, none of the HDL subclass proportions showed a significant association (Figure 3). Furthermore, the concentrations of the HDL subfractions or subclasses in mmol/L were not associated with ΔHR (see Supplementary Table S5). Detailed model estimates are provided in Supplementary Tables S4 and S5.

**Figure 4:**
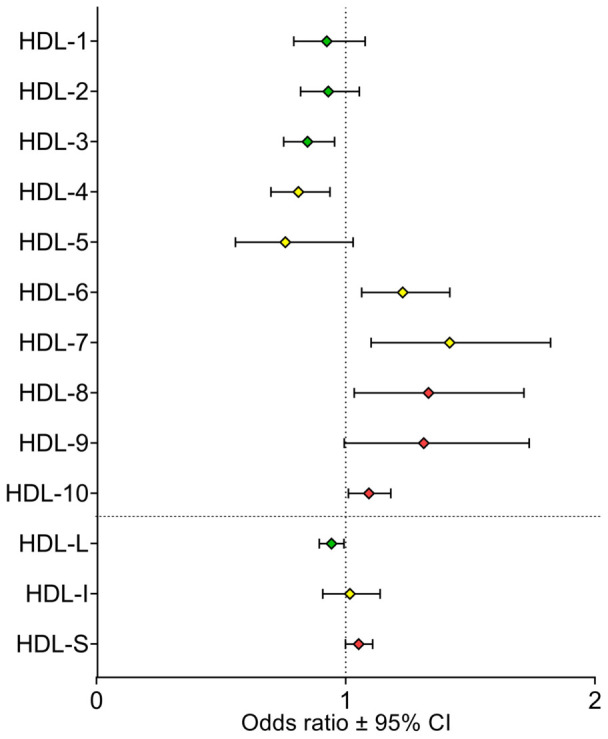
Binary logistic regression analysis of HDL subfraction and subclass percentages in relation to ΔHR_bi_. Colors represent the different HDL particle subclasses: green for large HDL (HDL-1 to HDL-3 and HDL-L), yellow for intermediate HDL (HDL-4 to HDL-7 and HDL-I), and red for small HDL (HDL-8 to HDL-10 and HDL-S). The vertical dotted line marks an odds ratio of 1.0. The horizontal dotted line separates the individual subfractions from the aggregated subclasses.

In the binary regression analysis, significant inverse associations with ΔHR_bi_ were observed for the proportions (in %) of HDL-3 (OR = 0.847; *p* = 0.017) and HDL-4 (OR = 0.810; *p* = 0.046). In contrast, significant positive associations were found for HDL-6 (OR = 1.229; *p* = 0.024), HDL-7 (OR = 1.417; *p* = 0.022), and HDL-8 (OR = 1.332; *p* = 0.044) (see Figure 4).

When HDL subfractions were analyzed based on their absolute concentrations (mmol/L), significant associations with ΔHR_bi_ were observed for HDL-4 (OR ≈ 0.000; *p* = 0.048) and HDL-5 (OR ≈ 0.000; *p* = 0.043), whereas no other subfractions or subclasses showed significant associations. For more details, see Supplementary Table S5.

### 3.5. Association of HDL Subfraction Profile with Resting Heart Rate (HR_rest_) and Heart Rate Immediately After Test (HR_aft_)

**Figure 5:**
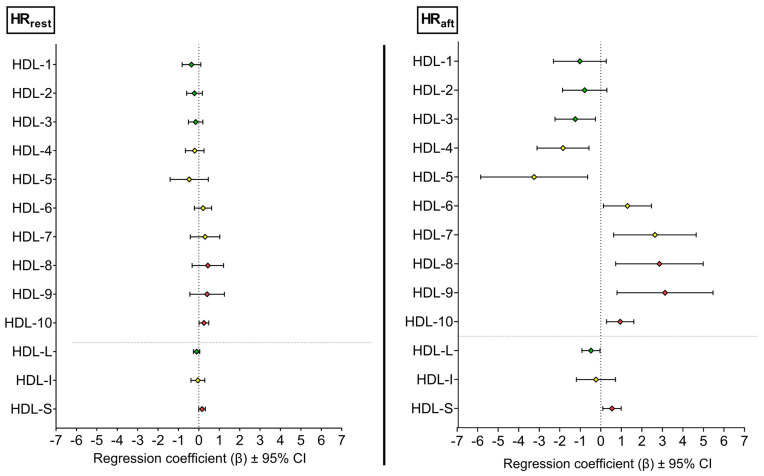
Association of HDL subfraction and subclass percentages with HR_rest_ and HR_aft_. Colors represent the different HDL particle subclasses: green for large HDL (HDL-1 to HDL-3 and HDL-L), yellow for intermediate HDL (HDL-4 to HDL-7 and HDL-I), and red for small HDL (HDL-8 to HDL-10 and HDL-S). The vertical dotted line marks a regression coefficient of zero. The horizontal dotted line separates the individual subfractions from the aggregated subclasses.

Composition of HDL subfractions and subclasses was not significantly associated with HR_rest_ (Figure 5).

HR_aft_ showed a significant inverse correlation for the percentages of HDL-3 (β = −1.278; *p* = 0.022), HDL-4 (β = −1.871; *p* = 0.040), and HDL-5 (β = −3.280; *p* = 0.020). On the other hand, HDL-6 (β = 1.269; *p* = 0.043), HDL-7 (β = 2.606; *p* = 0.018), HDL-8 (β = 2.826; *p* = 0.033), HDL-9 (β = 3.099; *p* = 0.025), HDL-10 (β = 0.913; *p* = 0.035), and the HDL-S subclass (β = 0.514; *p* = 0.038) demonstrated a significant positive correlation with the HR_aft_ (Figure 5). For the concentrations in mmol/L, see Supplementary Table S5.

### 3.6. Association of HDL Subfraction Profile with HR_5min_, HR_10min_, ΔHR_5min,_ and ΔHR_10min_

**Figure 6:**
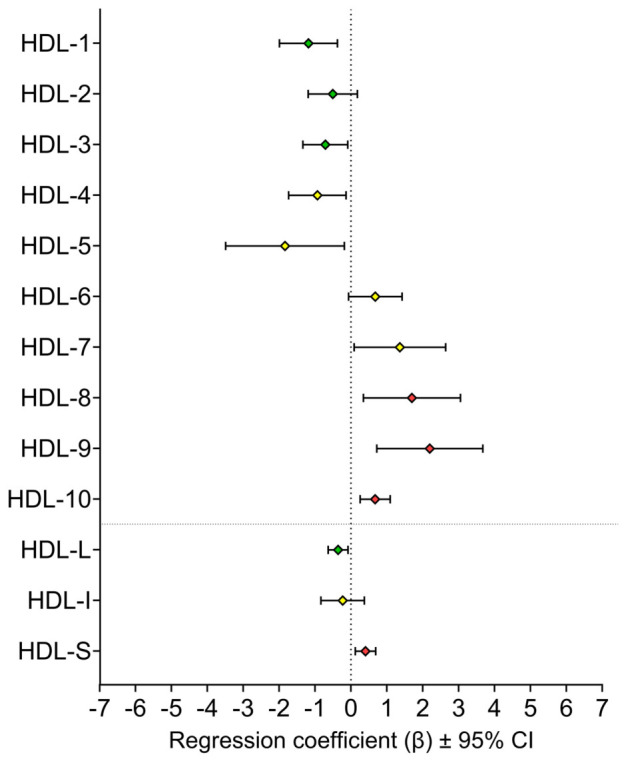
Association of HDL subfraction and subclass percentages with HR_5min._ Colors represent the different HDL particle subclasses: green for large HDL (HDL-1 to HDL-3 and HDL-L), yellow for intermediate HDL (HDL-4 to HDL-7 and HDL-I), and red for small HDL (HDL-8 to HDL-10 and HDL-S). The vertical dotted line marks a regression coefficient of zero. The horizontal dotted line separates the individual subfractions from the aggregated subclasses.

Figure 6 illustrates the significant negative associations of the proportions of HDL-1 (β = −1.185; *p* = 0.020), HDL-3 (β = −0.714; *p* = 0.043), HDL-4 (β = −0.936; *p* = 0.044), HDL-5 (β = −1.837; *p* = 0.041), and HDL-L (β = −0.358; *p* = 0.017) with HR_5min_. In contrast, the percentages of HDL-7 (β = 1.364; *p* = 0.045), HDL-8 (β = 1.698; *p* = 0.035), HDL-9 (β = 2.197; *p* = 0.013), HDL-10 (β = 0.675; *p* = 0.020), and the HDL-S subclass (β = 0.404; *p* = 0.015) were positively correlated with HR_5min_. However, only the concentrations of HDL-10 (β = 54.169; *p* = 0.048) and the HDL-S subclass (β = 32.210; *p* = 0.030) in mmol/L were significantly associated with HR_5min_ (Supplementary Table S5).

Only the percentage of HDL-1 and HDL-I significantly correlated with ΔHR_5min_ (β = 1.168, *p* = 0.040; and β = −0.719, *p* = 0.048, respectively), whereas the HDL-S subclass was significantly correlated with HR_10min_ (β = 17.333, *p* = 0.039). None of the subclasses or subfractions (expressed either in % or in mmol/L) were significantly associated with ΔHR_10min_ (see Supplementary Tables S4 and S5).

## 4. Discussion

This cross-sectional study investigated the association between high-density lipoprotein subfraction profiles and dynamic heart rate (HR) responses to exercise in a cohort of Hungarian adults. We found that HDL particle size distribution was associated with post-exercise heart rate responses. Larger HDL subfractions (HDL-3 to HDL-5) were associated with a more favorable post-exercise heart rate response, whereas smaller subfractions (HDL-7 to HDL-10) were associated with a less favorable response. These associations persisted after adjustment for traditional risk factors, while total HDL-C concentration showed no significant relationship with HR measures.

During acute physical exertion, the cardiovascular system undergoes significant hemodynamic stress. Immediate post-exercise recovery requires a coordinated cardiovascular system, a process involving vascular and endothelial adjustments [10,11,37].

HDL is a heterogeneous population of particles with potent vasoactive, antioxidant, and neuromodulatory properties. Large HDL particles are enriched in ApoA-I, paraoxonase-1 (PON1), and S1P [18,19,20]. These components have been linked to eNOS signaling and nitric oxide bioavailability, which may support vasodilation [20,38]. This increased vascular compliance optimizes the hemodynamic adjustments necessary for post-exercise heart rate deceleration [11]. In contrast, the accumulation of smaller, lipid-poor subfractions (HDL-7 to HDL-10) is frequently accompanied by reduced anti-inflammatory protection and diminished reverse cholesterol efflux capacity [16,39]. Small, dense HDL particles are highly susceptible to oxidative modification, which impairs their vascular reparative functions [40]. This diminished antioxidative capacity can lead to sustained systemic inflammation, which is known to blunt overall cardiovascular efficiency [41,42]. This may be reflected in the less favorable post-exercise heart rate responses observed in our cohort among individuals with predominantly smaller HDL particles. However, it is important to state that while the mentioned pathways involving ApoA-I, S1P, eNOS signaling, vascular compliance, oxidative stress, and inflammation are plausible, these mechanisms were not directly assessed in our study. Therefore, they should be interpreted as potential biological explanations rather than as findings of the study.

Previous epidemiological evidence provides indirect support for this lipid–cardiovascular axis. Elevated resting HR and delayed HR recovery have been linked to broader metabolic dysregulation. Williams et al. [43] reported an association between elevated resting HR and reduced concentrations of large HDL particles in sedentary men, while Shishehbor et al. [44] found that a higher triglyceride-to-HDL-C ratio was associated with impaired post-exercise HR recovery.

While most prior research has focused on static lipid parameters and resting HR, our study extends these observations by examining detailed HDL subfraction profiles in relation to a multi-stage, standardized post-exercise HR response obtained from the YMCA 3-min step test. Furthermore, our regression analyses are consistent with atherosclerosis literature, suggesting that specific HDL subfractions are linked not only to coronary artery disease but also to worse cardiorespiratory adaptability and subclinical vascular dysfunction. Xu et al. [45] demonstrated that elevated levels of small HDL subfractions independently predict coronary artery disease, while Asztalos et al. [46] observed an inverse relationship between large HDL particles and cardiovascular events.

In our cohort, lower ΔHR was associated with higher levels of HDL-C, ApoA-I, and more favorable lipid ratios, specifically a lower ApoB/ApoA-I ratio and a reduced LDL-C/HDL-C ratio. Previous studies have similarly reported that a more favorable lipid profile is associated with better cardiovascular outcomes in both observational and interventional settings [47,48,49,50].

Our findings suggest that younger individuals did not uniformly show more favorable heart rate responses than older adults. This unexpected pattern should be interpreted with caution. It may reflect sample characteristics or selection effects, such as survivor bias [51], whereby healthier individuals are more likely to be represented in older age groups. It may also partly reflect differences in habitual fitness and physical activity patterns across age groups [52,53,54,55]. Moreover, younger individuals in our study population were characterized by a less favorable cardiometabolic profile [56], which may have contributed in part to the unexpected age-related heart rate pattern.

In terms of practical implications, our findings suggest that HDL particle heterogeneity may provide additional biological context beyond that offered by standard lipid panels. Since total HDL-C concentration does not capture particle size distribution, relying on this conventional metric alone could lead to relevant differences in HDL composition being overlooked. These results support the need for further research into HDL subfraction patterns in relation to heart rate responses to exercise.

This study has several limitations. First, its cross-sectional design precludes causal inference, so the findings should be considered hypothesis-generating. Second, the final sample was drawn from a previously selected subgroup in which a substantial proportion of participants exhibited abnormal lipid profiles, which may limit generalizability to the broader adult population. Third, the YMCA step test is a simple submaximal field test rather than a maximal cardiopulmonary exercise test, and the observed heart rate responses may reflect multiple physiological determinants rather than autonomic regulation alone. Fourth, heart rate was assessed by manual palpation, which may introduce measurement variability. Fifth, the Lipoprint^®^ system classifies HDL by size rather than functional properties, and no universally accepted gold standard exists for HDL subclass analysis, which may affect comparability across studies [57]. Sixth, limited availability of data on menopausal status, supplements, and medications constrained adjustment for potential confounding; dietary and alcohol variables were not retained in the final models to preserve parsimony. Finally, physical activity was self-reported and may be affected by recall bias, while genetic and epigenetic factors were not assessed. Although the associations remained significant after Benjamini–Hochberg correction, external validation in larger, multiethnic cohorts is warranted.

Despite these limitations, this study offers notable strengths. It is among the first to directly bridge lipidomic heterogeneity with dynamic cardiorespiratory responses, significantly expanding the scope of basic lipid research. The inclusion of multiple heart rate metrics—resting, post-exercise, and recovery at 5 and 10 min—provides a nuanced assessment of cardiovascular adaptability. The use of a well-characterized, population-based cohort with standardized HDL subfraction analysis enhances the reproducibility and generalizability of the findings. Furthermore, the application of rigorous statistical adjustment and false discovery rate correction (Benjamini–Hochberg) strengthens the validity of the observed associations. By integrating lipidomic heterogeneity with physiological response patterns, the study contributes novel insights into the functional relevance of HDL particle distribution in cardiovascular regulation.

## 5. Conclusions

This cross-sectional study suggests that HDL particle size distribution, rather than total HDL-C concentration, may provide exploratory insight into heart rate dynamic responses to physical activity. Following submaximal physical exertion, specific HDL subfractions spanning the large and intermediate ranges (HDL-3 to HDL-5) were associated with a more favorable heart rate response, while smaller, lipid-poor HDL particles were associated with a less favorable response. Participants with more favorable post-exercise heart rate recovery also exhibited higher HDL-C and ApoA-I levels, along with lower ApoB/ApoA-I and LDL-C/HDL-C ratios, consistent with a more favorable lipid profile.

Taken together, these findings support HDL subfraction profiling as a potentially useful exploratory measure of exercise-related cardiovascular responses. Future studies incorporating mechanistic assays and longitudinal designs are warranted to confirm these associations and clarify the underlying biological pathways.

## Figures and Tables

**Table 1:** Demographic, clinical, and lifestyle characteristics across heart rate response to exercise (ΔHR) groups.

		Very Poor and Poor (*n* = 79)	Above Average/Average/Below Average (*n* = 133)	Good and Excellent (*n* = 92)	*p* for Trend
		Mean (95%CI)			
Age (years)		37.08 (34.69–39.47)	40.53 (38.29–42.76)	41.99 (39.39–44.59)	0.014 *
ΔHR (difference between HR immediately after exercise and resting HR)		66.68 (60.39–72.97)	26.96 (26.12–27.80)	15.88 (14.21–17.55)	<0.001 *
Maximum HR in percentage		80.52 (77.17–83.87)	59.22 (57.80–60.64)	49.52 (48.12–50.93)	<0.001 *
		Prevalence in % (95%CI)			*p* for trend
Roma		69.62 (58.91–78.92)	45.96 (37.56–54.35)	60.87 (50.96–70.38)	0.361
Women		70.89 (60.25–80.02)	59.40 (50.92–67.47)	77.17 (67.84–84.82)	0.287
Overweight/obesity (BMI ≥ 25 kg/m^2^)		74.68 (64.33–83.27)	63.16 (54.75–71.00)	56.52 (46.32–66.32)	0.021 *
Current smoker		56.96 (45.96–67.47)	51.13 (42.68–59.52)	55.43 (45.24–65.29)	0.890
Elevated diastolic blood pressure (≥90 mmHg)		18.99 (11.54–28.66)	13.53 (8.52–20.11)	5.43 (2.10–11.50)	0.007 *
Elevated systolic blood pressure (≥140 mmHg)		18.99 (11.54–28.66)	15.79 (10.36–22.96)	9.78 (4.95–17.09)	0.087
Anti-hypertension treatment		25.32 (16.73–35.67)	24.81(18.07–32.64)	20.65 (13.36–29.75)	0.459
Anti-diabetic treatment		7.59 (3.23–14.98)	7.52 (3.93–12.93)	5.43 (2.10–11.50)	0.563
Elevated fasting glucose level (≥7 mmol/L)		7.59 (3.23–14.98)	9.77 (5.59–15.68)	9.78 (4.95–17.09)	0.638
Leisure-time physical activity	0 MET-min/Week	39.24 (29.02–50.24)	29.32 (22.09–37.44)	20.65 (13.36–29.75)	0.026 *
	1–499 MET-min/Week	25.32 (16.73–35.67)	22.56 (16.09–30.20)	31.52 (22.71–41.47)	
	≥500 MET-min/Week	35.44 (25.57–46.36)	48.12 (39.75–56.58)	47.83 (37.82–57.97)	
Reduced HDL-C levels (<1.03 mmol/L in men and <1.29 mmol/L in women)		78.48 (68.50–86.42)	70.68 (62.56–77.91)	71.74 (61.97–80.16)	0.353

95%CI: 95% confidence interval; MET-min/week: metabolic equivalent task minutes per week; *: *p* < 0.05.

**Table 2:** Lipid and apolipoprotein profiles across heart rate response (ΔHR) categories following exercise.

	Very Poor and Poor (*n* = 79)	Above Average/Average/Below Average (*n* = 133)	Good and Excellent (*n* = 92)	*p* for Trend
	Mean (95%CI)			
TG (mmol/L)	1.75 (1.49–2.01)	1.63 (1.46–1.81)	1.53 (1.31–1.76)	0.241
Total Cholesterol (mmol/L)	4.71 (4.46–4.96)	4.69 (4.53–4.84)	4.54 (4.30–4.77)	0.147
HDL-C (mmol/L)	1.12 (1.04–1.19)	1.17 (1.11–1.23)	1.21 (1.14–1.28)	0.014 *
LDL-C (mmol/L)	3.06 (2.82–3.29)	2.95 (2.81–3.08)	2.82 (2.62–3.01)	0.144
TG/HDL-C ratio	4.14 (3.41–4.87)	3.75 (3.22–4.29)	3.35 (2.75–3.94)	0.063
Total Cholesterol/HDL-C ratio	4.53 (4.18–4.87)	4.29 (4.05–4.53)	3.99 (3.70–4.28)	0.010 *
LDL-C/HDL-C ratio	2.95 (2.67–3.22)	2.71 (2.53–2.89)	2.50 (2.27–2.73)	0.008 *
ApoA-I (g/L)	1.33 (1.28–1.37)	1.39 (1.35–1.43)	1.41 (1.36–1.46)	0.018 *
ApoB (g/L)	1.06 (0.99–1.13)	1.03 (0.99–1.08)	0.98 (0.91–1.05)	0.066
ApoB/ApoA-I ratio	0.82 (0.76–0.88)	0.77 (0.73–0.81)	0.72 (0.66–0.77)	0.009 *
LDL-C/ApoB ratio	1.11 (1.08–1.13)	1.11 (1.09–1.13)	1.11 (1.08–1.13)	0.906

95%CI: 95% confidence interval; TG, triglyceride; HDL-C, high-density lipoprotein cholesterol; LDL-C, low-density lipoprotein; ApoA-I, apolipoprotein A1; ApoB, apolipoprotein B; *: *p* < 0.05.

## Data Availability

The dataset(s) supporting the conclusions of this article are available upon request from the study coordinators, Prof. Róza Ádány (adany.roza@med.unideb.hu) and Dr. Péter Pikó (piko.peter@med.unideb.hu), due to data protection and ethical concerns.

## Supplementary Materials

The following supporting information can be downloaded at https://www.mdpi.com/article/10.3390/biology15131051/s1.

## Abbreviations

The following abbreviations are used in this manuscript:

ApoA-I	Apolipoprotein A-I
ApoB	Apolipoprotein B
ASCVD	Atherosclerotic Cardiovascular Disease
BMI	Body Mass Index
BP	Blood Pressure
CI	Confidence Interval
EHIS	European Health Interview Survey
eNOS	Endothelial Nitric Oxide Synthase
HDL	High-Density Lipoprotein
HDL-C	High-Density Lipoprotein Cholesterol
HDL-L	Large High-Density Lipoprotein Subclass
HDL-I	Intermediate High-Density Lipoprotein Subclass
HDL-S	Small High-Density Lipoprotein Subclass
HG	Hungarian General Population
HR	Heart Rate
HR_5min_	Heart Rate 5 Min Post-Exercise
HR_10min_	Heart Rate 10 Min Post-Exercise
HR_aft_	Heart Rate Immediately After Exercise
HR_max_	Age-Related Maximum Heart Rate
HR_rest_	Resting Heart Rate
HRV	Heart Rate Variability
IPAQ	International Physical Activity Questionnaire
LDL-C	Low-Density Lipoprotein Cholesterol
MET-min/week	Metabolic Equivalent of Task minutes per week
NO	Nitric Oxide
OR	Odds Ratio
PON1	Paraoxonase-1
S1P	Sphingosine-1-phosphate
TC	Total Cholesterol
TG	Triglycerides
VIF	Variance Inflation Factor
ΔHR	Delta Heart Rate (Difference between HR_aft_ and HR_rest_)
ΔHR_Bi_	Binary Delta Heart Rate (Dichotomized heart rate response)
